# Early Intervention Developmental Programming and Childhood Academic Outcomes

**DOI:** 10.1001/jamanetworkopen.2025.55890

**Published:** 2026-02-09

**Authors:** Jeanette A. Stingone, Katharine H. McVeigh, Lidiya Lednyak

**Affiliations:** 1 Department of Epidemiology, Columbia University Mailman School of Public Health, New York, New York; 2 Bureau of Early Intervention, NYC Department of Health and Mental Hygiene–City of New York, New York, New York

## Abstract

**Importance:**

National monitoring surveys indicate that developmental disabilities among US children constitute a substantial public health issue. While scientific literature documents the benefits of targeted, developmental interventions, there has been less study of formal early intervention (EI) services provided through Part C of the Individuals With Disabilities Education Act.

**Objective:**

To assess the population-level utilization of the New York City EI program and estimate the association between receipt of EI services before 3 years of age and academic achievement later in childhood.

**Design, Setting, and Participants:**

This retrospective cohort study was performed within an administrative data linkage of public health and educational data systems in New York City, with records from January 1, 1994, to December 31, 2007. Participants included children born in New York City between January 1, 1994, and December 31, 1998, who attended public elementary school for third grade. Analyses were conducted from January 1, 2023, to December 31, 2024.

**Exposure:**

Any use of EI services from birth through 3 years of age.

**Main Outcomes and Measures:**

The primary outcomes were standardized test scores in math and English language arts (ELA) in third grade. After propensity score matching, linear and log-binomial regression were used to estimate differences in standardized test scores and incidence ratios of meeting test-based standards, comparing individuals who did and did not receive EI services.

**Results:**

The study population consisted of 214 370 children with records through third grade. Of the 13 022 children who had received EI services (6.1%) before 3 years of age, 8516 (65.4%) were male (mean [SD] gestational age, 37.5 [3.8] weeks). When examining third grade standardized test scores, higher absolute test scores were observed among children who received EI in ELA (estimate, 0.045; 95% CI, 0.021-0.069) and greater incidence of meeting test-based standards in both math (incidence ratio, 1.08; 95% CI, 1.06-1.10) and ELA (incidence ratio, 1.09; 95% CI, 1.07-1.12) when comparing propensity score–matched samples. Evidence of heterogeneity was observed, as individuals who required special education, those from households with lower socioeconomic status, and those born to immigrant mothers had greater test score benefits associated with EI.

**Conclusions and Relevance:**

The findings of this cohort study suggest that EI services for children younger than 3 years with moderate to severe developmental delays or disabilities had tangible academic benefits later in childhood. Future research should investigate the implementation of EI services among individuals with different diagnoses and developmental delays to determine the most beneficial service plans for children with differing needs.

## Key Points

**Question:**

Is receipt of early intervention (EI) services before 3 years of age associated with performance on standardized testing among children in third grade?

**Findings:**

In this cohort study including 13 022 children who received EI, receipt of EI services before 3 years of age was associated with greater test *z* scores in English language arts and increased incidence of meeting curricula-based test standards in both math and English language arts in third grade.

**Meaning:**

These findings suggest that an EI program may have tangible academic benefits for children who have moderate to severe developmental delays or disabilities and are living in a large urban center.

## Teaser

This cohort study assesses the association between receipt of early intervention services via the Individuals With Disabilities Education Act and subsequent academic achievement among children in New York City who have developmental delays or disabilities.

## Introduction

From 2009 to 2017, the prevalence of diagnosed developmental disabilities among children aged 3 to 5 years was 10.6%.^1^ Children born weighing less than 2500 g, boys, children with public insurance, children with mothers with less than a college education, and children living in low-income households were at highest risk of being diagnosed with developmental disabilities. These national data underscore the need for ongoing monitoring and resource allocation to support affected individuals and their families.^1^

Randomized and quasi-randomized controlled trials provide evidence that interventions for children with developmental diagnoses and delays can have beneficial effects on development.^2,3,4,5,6^ Orton et al^2^ conducted a review of intervention programs for preterm infants in the first year of life and found benefits on cognitive outcomes to preschool, but results were mixed later in childhood. A meta-analysis on parent-implemented early language interventions for children with developmental delays^4^ found an overall beneficial association with communication and language interactions. Results highlight the benefit of intervening early in childhood, potentially before specific diagnoses can be determined. The rapid pace of neural development during infancy underscores the importance of early intervention (EI), as the brain’s capacity for change diminishes with age.^7^ Neuroscience research^8,9^ suggests that interventions during early childhood yield superior outcomes compared with remediation later in life.

There has been less study of the effects of formal programming and services provided through Part C of the Individuals With Disabilities Education Act (IDEA). Mandated in 1986, Part C established EI programs for infants and toddlers with disabilities or delayed development as a critical component of population-level interventions to improve early childhood development in the US.^10^ The US Department of Education’s Office of Special Education Programs administers Part C to states, territories, and eligible jurisdictions and monitors compliance and outcomes. New York State began implementing this mandate in July 1993.^11^ As it is not possible to randomize children to services ensured by legislation, observational studies have been used to quantify the impact of receiving services. Findings from observational studies face challenges related to exchangeability in comparison groups and residual confounding. The National Early Intervention Longitudinal Study^12^ found infants and toddlers receiving Part C services showed notable improvements in motor, social, and cognitive functioning and the acquisition of age-appropriate skills. More recent analysis of state-level data^13,14^ further substantiates these findings, reporting significant developmental gains among children enrolled in EI. However, these analyses examined broad developmental outcomes shortly after receipt of EI services and relied on preintervention and postintervention comparisons, without a comparison group that did not receive services. Constructing an appropriate comparison group is challenging, as service receipt and academic achievement are linked to multiple shared characteristics^15,16^ that must be adequately controlled for in analyses.

Given clinicians’ key role in identifying and referring children with delays,^17^ health care practitioners must be aware of EI services and confident in their effectiveness.^18^ Strong methodologic research documenting the effectiveness and continued need for these partially federally supported programs is especially important in light of recent proposals to cut funding for the Department of Education and Medicaid.^19^ The Longitudinal Study of Early Development (LSED), conducted by the New York City Health Department, aimed to assess the population-level utilization of the NYC EI program and estimate the association between receipt of Part C EI services before 3 years of age and subsequent academic achievement in third grade.^20^

## Methods

### Study Design and Population

LSED is an administrative data linkage of records for children born or living in NYC from January 1, 1994, to December 31, 2004, with data available through December 31, 2007.^20^ Records across the NYC birth and death registries, childhood lead surveillance data, eligibility and use data from the EI program, and third-grade standardized test scores, special education, and other administrative data from the Department of Education were matched using probabilistic data linkage. For this analysis, we included individuals born between January 1, 1994, and December 31, 1998, to mothers living in NYC. Included individuals attended a public school in NYC for third grade and had scores on the third grade standardized tests prior to December 31, 2007 (eFigure 1 in Supplement 1). Children who died prior to third grade were excluded.

All study-related activities were approved by Institutional Review Boards at Columbia University and the NYC Health Department. The study was approved with a waiver of informed consent as a secondary analysis of data with personal health information removed, reflecting minimal risk to participants and no way to conduct the research without the waiver. The study followed the Strengthening the Reporting of Observational Studies in Epidemiology (STROBE) reporting guideline.

### Outcomes of Interest: Standardized Test Scores

Standardized tests in math and English language arts (ELA) were administered to third grade students as part of standard educational protocols in the NYC public schools. We standardized raw scores using year of birth as a proxy for test year to calculate *z* scores. Each year, raw scores were also converted into categories of exceed, meet, do not meet, or exceedingly do not meet curricula-based standards. We collapsed this multicategory variable into a binary variable that contrasted those who met or exceeded curricula-based standards with those who did not meet standards.

### Definition of EI Services

We defined EI services as documented receipt of any EI core services during the first 36 months of life. Core services include occupational therapy, physical therapy, speech therapy, or special instruction. To receive EI services, children were first referred to the NYC EI Program by parents, caregivers, pediatricians, or other health care or educational professionals. The child then received a multidisciplinary evaluation to determine eligibility. If a child’s evaluation demonstrated a substantial delay in 1 of 5 areas of development or a moderate delay in at least 2 areas or if the child had a single qualifying condition with a high probability of developmental delay or disability, a service plan was developed, and family concerns, priorities, and resources were discussed. Outcomes were developed, and services were authorized and then implemented, ensuring that the services were family driven. Children who were either ineligible for services or authorized for services but had no documented receipt of services were grouped with the individuals who were never referred to EI.

### Construction of Propensity Score

We constructed propensity scores using the random forest algorithm to indicate an individual’s probability of receiving EI services, based on a set of measured variables. Variables used to construct the propensity score (Box), including maternal characteristics and child characteristics at birth and in early childhood, were identified via directed acyclic graph analysis to determine variables that could induce potential confounding and/or were associated with the achievement on standardized tests.^21^ Maternal race and ethnicity were extracted from the birth certificate and reflect self-reporting using prespecified categories, including Black, Latina, White, and other race or ethnicity (consisting of American Indian or Alaska Native, Asian, and Native Hawaiian or Other Pacific Islander). Within this analysis, race and ethnicity were used as proxies for social and structural factors that contribute to disparities in accessing early intervention services.

Box. **Variables Used to Construct the Propensity Score to Facilitate Exchangeability When Comparing Individuals Who Have and Have Not Received Early Intervention Services**

**Maternal characteristics during pregnancy and/or at birth**

Age
Race and ethnicity
Nativity
Occupation
Educational attainment
Borough of residence
Neighborhood deprivation index of residential census tract calculated from 2000 US Census data
Borough of residence
Timing of prenatal care
Insurance status
Tobacco and alcohol use
Prepregnancy weight

**Child characteristics at birth**

Sex
Year of birth
Season of birth
Being part of a multiple gestation
Presence of congenital anomalies
Birth weight
Gestational age

**Child characteristics in early childhood**

Residential stability (total number of residential addresses)
Eligibility for free lunch programs
Number of school absences in third grade
Need for special education services^a^
Maximum blood lead level

^a^
Defined as having an individual education plan at 3, 4, and/or 5 years of age and/or third grade.

Related work showed results were consistent with using logistic regression^22^; thus, only random forest was assessed in this analysis. We constructed a forest of 500 trees using bootstrapping with one-third of the variables eligible for random selection at each potential node split. The minimum node size was set to 5, but we did not restrict the number of terminal nodes. Multiple imputation using multiple chain equations was performed to impute missing data on variables needed to construct the propensity score.^23^ We performed 5 imputations and repeated the subsequent analytic pipeline for each imputation, combing results using Rubin rules.^24^

### Statistical Analysis

Analyses were conducted from January 1, 2023, and December 31, 2024. Using propensity score matching, we matched each EI recipient with as many as 3 nonrecipients whose propensity scores were within 0.1 SDs. To isolate the association with EI, we matched on special education, defined as having an individual education plan (IEP) at ages 3, 4, and/or 5 years and/or in third grade. An IEP indicated a child was determined to need special education services but did not confirm service receipt. We confirmed overlap in propensity scores before matching and then calculated the average standardized absolute mean difference for all variables before and after matching to assess how matching improved exchangeability.

General linear models were constructed to estimate associations between EI and test *z* scores, while log-binomial models were constructed to estimate the incidence ratio when using the binary indicator of meeting test-based standards as the outcome. To determine whether the association was consistent across the distribution of propensity scores, we stratified by propensity score and reran models within each quintile-defined stratum, using quintiles defined by individuals receiving EI.^25^ Because the goal was to estimate the magnitude and precision of the association between receipt of EI and test scores, we did not perform null hypothesis testing and instead report the estimates and associated 95% CIs rather than *P* values.^26^

To estimate the association of EI and test scores within subpopulations, we stratified our population into subgroups defined by demographic and educational characteristics and replicated our full analytic pipeline (ie, propensity score construction through modeling) within each subgroup. Estimating separate propensity scores within subgroups, rather than including an interaction term in the main outcome model, was pursued to ensure covariate balance would be maintained within the subgroups.^27^

To account for potential confounding due to neurodevelopmental need, we conducted a sensitivity analysis focusing only on individuals who were determined to need special education services after 3 years of age. Special education referral may serve as an imperfect proxy for underlying neurodevelopmental need. We also looked at patterns of special education services hypothesized to show weaker associations between EI and test scores. For example, receiving only speech therapy would be limited to individuals with only a communication disorder, such as stuttering, impaired articulation, or a language or voice impairment, which may not affect performance on written examinations. Analyses were performed in R, version 4.4.1 (R Project for Statistical Computing) using the mice,^23^ randomForest,^28^ and MatchIt^29^ packages.

## Results

The study population consisted of 214 370 children, of whom 13 022 had ever received EI services (6.1%). Of these, 4506 children (34.6%) were female and 8516 (65.4%) were male; maternal race and ethnicity included 4536 (348%) who were Black, 5506 (42.3%) who were Latina, 2255 (17.3%) who were White, and 725 (5.6%) who were other race or ethnicity. Table 1 presents the demographic characteristics of the study population, reflecting known patterns: boys, children with younger gestational ages, and those from neighborhoods with lower socioeconomic levels were more likely to receive EI services. The average standardized absolute mean difference across variables was reduced to 0.04 from 0.17, suggesting adequate balance after matching. Substantial overlap in propensity scores between groups allowed retention of most of the initial sample after matching (eFigure 2 in Supplement 1).

**Table 1.** Demographic Characteristics Stratified by Receipt of EI Services Before and After Propensity Score Matching and Imputation^a^

Characteristic	Before propensity-score matching and imputation		After propensity-score matching and imputation	
	Received EI services (n = 13 022)	Did not receive EI services (n = 201 348)	Received EI services (n = 12 432)	Did not receive EI services (n = 34 459)
Gestational age, mean (SD), wk	37.5 (3.8)	38.9 (2.0)	37.9 (3.3)	38.4 (2.8)
Sex, No. (%)				
Female	4506 (34.6)	103 194 (51.3)	4266 (34.3)	13 024 (37.8)
Male	8516 (65.4)	98 154 (48.7)	8166 (65.7)	21 435 (62.2)
Maternal race and ethnicity, No. (%)				
Black	4536 (34.8)	69 898 (34.7)	4268 (34.3)	12 046 (35.0)
Latina	5506 (42.3)	74 502 (37.0)	5322 (42.8)	14 156 (41.1)
White	2255 (17.3)	34 016 (16.9)	2143 (17.2)	5951 (17.3)
Other^b^	725 (5.6)	22 932 (11.4)	699 (5.6)	2306 (6.7)
Mother born outside the US, No. (%)	5621 (43.2)	102 181 (50.7)	5376 (43.2)	14 776 (42.9)
Paternal information on birth certificate, No. (%)	8913 (68.4)	149 071 (74.0)	8529 (68.6)	24 186 (70.2)
Need for special education services, No. (%)^c^	8728 (67.0)	31 623 (15.7)	8192 (65.9)	21 978 (63.8)
Maternal age, mean (SD), y	28.0 (6.5)	27.3 (6.4)	27.9 (6.5)	27.6 (6.6)
Maternal educational level, mean (SD), y	11.8 (2.7)	11.8 (2.6)	11.8 (2.7)	11.7 (2.7)
Neighborhood Deprivation Index, mean (SD)^d^	0.50 (2.05)	0.40 (2.03)	0.51 (2.05)	0.48 (2.11)

Abbreviation: EI, early intervention.

^a^ After imputation represents a single imputation of analytic population with both tests (math and English language arts) after propensity score matching. After imputation, there were no missing data.

^b^ Includes American Indian or Alaska Native, Asian, and Native Hawaiian or Other Pacific Islander.

^c^ Need for special education services was defined as having an individual education plan at 3, 4, and/or 5 years of age and/or third grade.

^d^ Scores range from −5.6 to 5.7, with higher scores indicating greater neighborhood deprivation.

The Figure provides the estimated difference in third grade test scores by receipt of EI and incidence of meeting test-based standards in the propensity score–matched group and stratified by quintiles of propensity score. When stratifying by propensity score, we consistently see the magnitude of associations was greater when examining individuals who had a greater probability of receiving EI services. We observed beneficial associations between EI and ELA *z* score and greater incidence of meeting standards on both math and ELA tests among children who received EI services. In contrast, we saw a generally null association between EI and math *z* scores when examining the full population. Higher absolute test scores were observed in ELA (estimate, 0.045; 95% CI, 0.021-0.069) and greater incidence of meeting test-based standards in both math (incidence ratio, 1.08; 95% CI, 1.06-1.10) and ELA (incidence ratio, 1.09; 95% CI, 1.07-1.12) among children with a greater probability of receiving EI services when comparing propensity score–matched samples.

**Figure.**
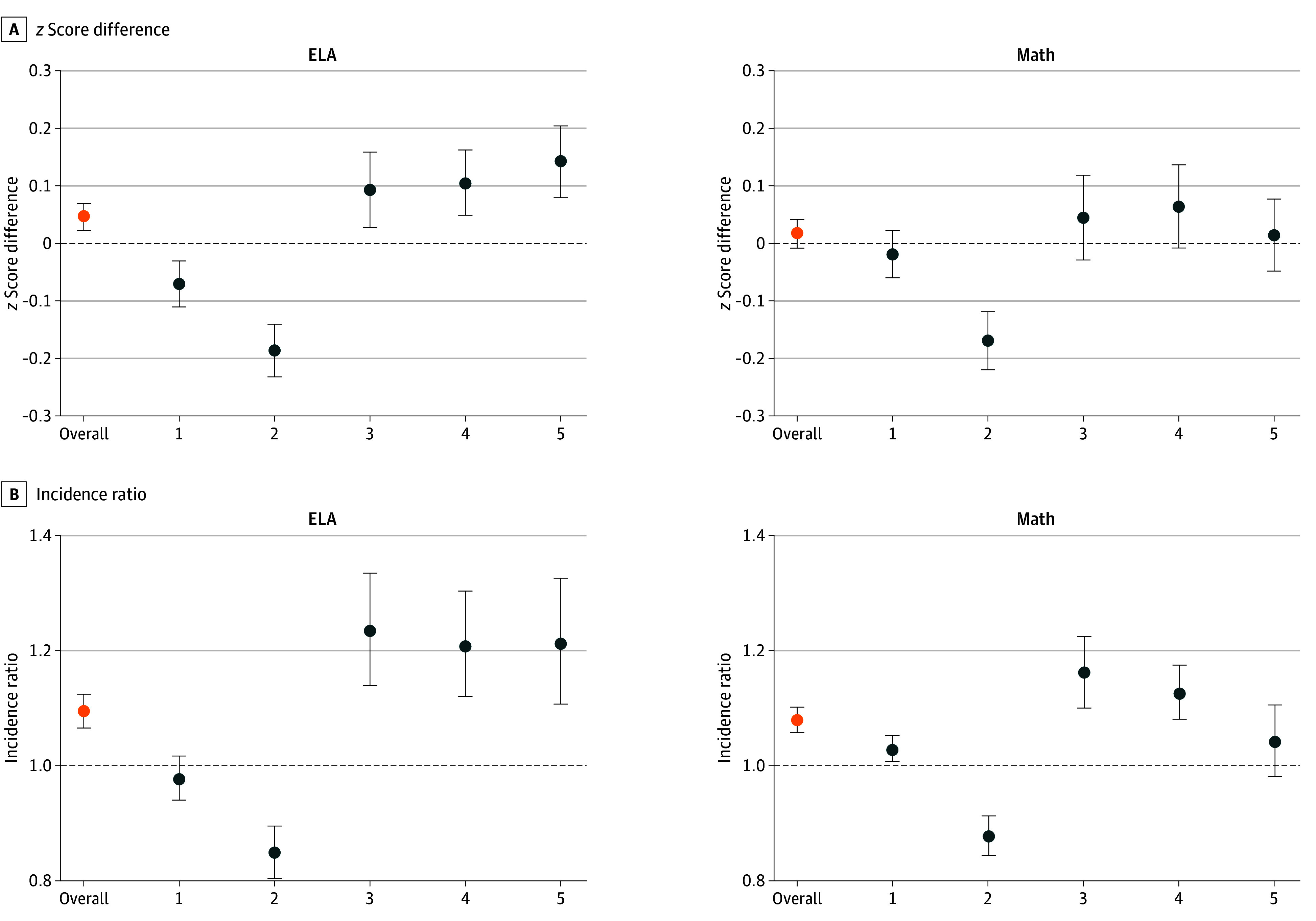
Estimated Differences in z Scores and Incidence Ratios Associated With Receipt of Early Intervention Services Data from the New York City Longitudinal Study of Early Development are shown overall and stratified by quintile (Q) of propensity score for English language arts (ELA; left) and math (right) standardized test scores. Higher quintiles indicate individuals with higher estimated probability (eg, propensity) of receiving early intervention services. Error bars indicate 95% CIs.

Table 2 provides the results within subpopulations defined by features used to construct the propensity score that were hypothesized to potentially modify the association between EI and outcomes. The largest estimates were observed when restricted to the subpopulation of individuals who were determined to need special education services. Among children who were determined to need special education services, children who had received EI had an incidence ratio 1.28 (95% CI, 1.23-1.34; 28% greater likelihood) for meeting test-based standards in ELA and 1.17 (95% CI, 1.13-1.20; 17% greater likelihood) for meeting test-based standards in math when compared with individuals who had not received EI services. Among the subpopulation that did not require special education later in childhood, we did not see differences in math and ELA test scores between those who received EI and those who did not. Among children who received IEPs by 5 years of age, there was no difference in the proportion of children who remained enrolled through third grade by receipt of EI services (73.2% for no EI vs 71.0% for EI). However, among children who did not receive IEPs by 5 years of age, children who received EI services were less likely to enroll in public school for third grade (87.1% for no EI vs 69.5% for EI).

**Table 2.** Estimated Difference in *z* Score and Incidence Ratios Associated With Receipt of Early Intervention Services for ELA and Math Standardized Tests Within Select Subpopulations^a^

Variable	No. of participants	Estimate (95% CI)		Incidence ratio (95% CI)	
		Math *z* score	ELA *z* score	Math met standards	ELA met standards
Determined need for special education^b^	7580	0.056 (0.026 to 0.085)	0.112 (0.085 to 0.139)	1.17 (1.13 to 1.20)	1.28 (1.23 to 1.34)
No determined need for special education	4195	−0.026 (−0.062 to 0.011)	−0.05 (−0.084 to −0.016)	1.01 (0.99 to 1.03)	0.97 (0.95 to 1.00)
Types of special education^c^					
Any physical therapy^d^	1479	0.048 (−0.051 to 0.147)	0.046 (−0.050 to 0.142)	1.10 (1.01 to 1.19)	1.04 (0.92 to 1.17)
Occupational therapy, no physical therapy^d^	195	0.004 (−0.290 to 0.297)	0.089 (−0.141 to 0.319)	1.19 (0.90 to 1.57)	1.07 (0.71 to 1.62)
Speech therapy only^d^	264	−0.004 (−0.206 to 0.198)	0.020 (−0.159 to 0.199)	0.93 (0.76 to 1.13)	0.94 (0.68 to 1.32)
Maternal race and ethnicity					
Asian and other^e^	640	0.015 (−0.08 to 0.111)	−0.001 (−0.108 to 0.106)	1.03 (0.98 to 1.07)	1.07 (0.99 to 1.15)
Black	3989	−0.006 (−0.046 to 0.035)	0.056 (0.017 to 0.096)	1.09 (1.05 to 1.14)	1.12 (1.06 to 1.18)
Latina	5107	0.057 (0.024 to 0.091)	0.073 (0.038 to 0.107)	1.13 (1.10 to 1.16)	1.15 (1.10 to 1.21)
White	2015	−0.027 (−0.082 to 0.029)	−0.025 (−0.085 to 0.034)	1.01 (0.98 to 1.04)	1.02 (0.98 to 1.07)
Nativity of Latina mothers					
Born outside the US	2763	0.093 (0.049 to 0.136)	0.108 (0.065 to 0.150)	1.15 (1.11 to 1.18)	1.18 (1.11 to 1.25)
Born in the US	2276	0.017 (−0.035 to 0.070)	0.033 (−0.020 to 0.086)	1.10 (1.05 to 1.15)	1.12 (1.04 to 1.22)
Insurance					
Medicaid	8005	0.021 (−0.007 to 0.049)	0.059 (0.031 to 0.086)	1.10 (1.08 to 1.13)	1.13 (1.08 to 1.18)
No Medicaid	3771	0.018 (−0.03 to 0.066)	0.028 (−0.018 to 0.074)	1.05 (1.02 to 1.07)	1.07 (1.03 to 1.11)
Maternal educational level					
Less than high school	4142	0.026 (−0.013 to 0.065)	0.052 (0.015 to 0.088)	1.12 (1.08 to 1.16)	1.13 (1.07 to 1.19)
High school to some college	6406	0.020 (−0.014 to 0.053)	0.051 (0.020 to 0.082)	1.08 (1.05 to 1.10)	1.10 (1.06 to 1.14)
College and higher	1215	−0.025 (−0.098 to 0.487)	−0.061 (−0.135 to 0.013)	1.01 (0.98 to 1.04)	1.01 (0.96 to 1.06)
Preterm birth	2209	−0.108 (−0.158 to −0.057)	−0.043 (−0.092 to 0.006)	0.97 (0.93 to 1.01)	0.99 (0.93 to 1.05)
Birth weight, g					
<1500^d^	782	−0.228 (−0.331 to −0.125)	−0.104 (−0.205 to −0.004)	1.05 (0.79 to 0.96)	0.88 (0.78 to 1.00)
1500-2500^d^	1619	−0.04 (−0.107 to 0.027)	−0.013 (−0.089 to 0.063)	1.07 (1.00 to 1.14)	1.04 (0.95 to 1.14)
>2500	9823	0.049 (0.024 to 0.074)	0.058 (0.032 to 0.084)	1.10 (1.08 to 1.12)	1.11 (1.08 to 1.14)
<2500^d^	2478	−0.089 (−0.145 to −0.034)	−0.038 (−0.093 to 0.017)	1.01 (0.96 to 1.06)	1.00 (0.93 to 1.07)

^a^ Sample sizes refer to the number of individuals within a subpopulation who received early intervention (EI) services. Each EI recipient was then matched with as many as 3 nonrecipients. Data are from the New York City Longitudinal Study of Early Development study.

^b^ Defined as having an individual education plan at 3, 4, and/or 5 years of age and/or third grade.

^c^ Determined by specific types of services included in the individual education plan. These subgroups are not exhaustive and represent subgroups of particular interest due to the hypothesis that these groups may see less of an association between EI and test scores.

^d^ Fewer than 3 EI nonrecipients were able to be matched to each EI recipient.

^e^ Includes American Indian or Alaska Native and Native Hawaiian or Other Pacific Islander.

Children of Latina mothers, especially those born to mothers outside the US, also had associations between EI and math (estimate, 0.057; 95% CI, 0.024-0.091) and ELA (estimate, 0.073; 95% CI, 0.038-0.107) test scores and meeting test-based standards (incidence ratios, 1.13 [95% CI, 1.10-1.16] and 1.15 [95% CI, 1.10-1.21], respectively). Children whose mothers were enrolled in Medicaid at the time of delivery (estimate for math, 0.021 [95% CI, −0.007 to 0.049]; estimate for ELA, 0.059 [95% CI, 0.031-0.086]) and children whose mothers had lower educational attainment (estimate for math, 0.026 [95% CI, −0.013 to 0.065]; estimate for ELA, 0.052 [95% CI, 0.015-0.088]) demonstrated greater benefits than other children from participating in EI. However, we still observed increased incidence of meeting test-based standards among children whose mothers did not have Medicaid insurance (incidence ratio for math, 1.05 [95% CI, 1.02-1.07]; incidence ratio for ELA, 1.07 [95% CI, 1.03-1.11]). We observed lower test scores among preterm children (estimate for math, −0.108 [95% CI, −0.158 to −0.057]; estimate for ELA, −0.043 [95% CI, −0.092 to 0.006]) and those with low birth weight (estimate for math, −0.228 [95% CI, −0.331 to −0.125]; estimate for ELA, −0.104 [95% CI, −0.205 to −0.004]) who received EI, although difficulty in identifying comparators suggests caution when interpreting these results.

## Discussion

In this cohort study, receipt of EI services before 3 years of age was associated with greater ELA test *z* scores and increased incidence of meeting curricula-based test-standards in math and ELA in third grade. Notably, among children with an indicated need for special education after 3 years of age, outcomes were better in children who had received EI services. We also observed heterogeneity in our findings, with children of Latina mothers, especially mothers born outside of the US, and children born to mothers with lower educational attainment and using public insurance having a greater association between EI and test-based achievement in math and ELA.

These results are consistent with prior literature that reports beneficial associations between early-life developmental interventions for children and subsequent academic performance. Elbum and Celmili-Aksoy^13^ observed improvements in children’s performance on a validated assessment for developmental functioning and found approximately 25% scored within age expectations across all domains. We found larger percentages of children performing consistently with expectations by third grade, with more than 50% of children meeting curricular-based test standards for math, but closer to 40% for ELA. Average test *z* scores within our matched study population were lower than the average within the full third grade population, suggesting continued need for supportive services for some children. Prior studies^12,13^ examined developmental and broader cognitive outcomes, making it difficult to directly compare our findings on math and ELA performance. The larger magnitudes observed for incidence ratios compared with *z* score differences reflect a common feature of normally distributed outcomes. Small shifts in the mean of the distribution (ie, difference in *z* score) correspond to a larger change in the proportion falling below a threshold (ie, proportion below the *z* score that corresponds to failing to meet standards) because tail probabilities change exponentially with distance from the mean.^30^

A previous sequence analysis within LSED revealed that children who received both EI and special education had better outcomes than individuals who only received one.^31^ Continuity of services in the presence of need is well-established as being beneficial for a child’s development, and a number of barriers, including lack of resources, changing diagnoses, and communication barriers, have been documented.^16,32,33^ Our findings support the need to ensure smooth transitions between EI programs and enrollment in school-based programs for children who demonstrate educational disabilities when they age out of EI.

Associations between EI and test scores varied within subpopulations defined by demographic characteristics. It is possible children of Latina mothers born outside the US may show more benefit from EI due to greater exposure to English-led instruction, simultaneously improving language skills. However, since its inception, the practice has been to provide family-centered EI services, which would have included working with parents in the language that is spoken in their home. In addition, multilingual EI service professionals have been available in NYC since at least the late 1990s.^34^ The lack of benefit of EI among children of parents with higher educational attainment has been observed in prior research.^35^ This could be explained by parents with higher educational attainment having greater resources to provide stimulation and enrichment, even without EI. In addition, research suggests that household exposure to complex language can benefit early language development.^36^ This aspect of EI services may already be met in households of highly educated parents, limiting the benefit to further development.

Our findings suggesting no benefit among children with very low birth weight and children born preterm should be interpreted with caution. Very low birth weight is an autoeligible condition, and it is unclear why individuals with an autoeligible condition would not subsequently receive services. It is possible children received privately accessed interventions, including hospital rehabilitative services and long-term care, which would not be recorded within these records. Different study designs, such as regression discontinuity approaches, may be needed to study EI and test scores in individuals with autoeligible conditions.

Heterogeneity in associations across propensity score strata may reflect effect measure modification, nonlinearity, variability in treatment need or effectiveness, or residual imbalance. Our subgroup analyses suggest potential effect measure modification. However, the lack of data on need for services may contribute to residual confounding. For example, children in the lower propensity score strata may have had less need for EI, explaining the observed null and inverse associations between test scores and EI. Future studies should be powered to explore variation in associations across subpopulations to inform more targeted policies and practices.

### Strengths and Limitations

Combining a large population-based data linkage with propensity score methods is a methodologic strength of the study. However, confounding due to unmeasured variables is still possible. Our findings for children of Latina mothers may be due to language, but we do not have any data on language spoken in the home, the child’s language skills, or the language used to provide EI services. We do not have any direct measures of neurodevelopment in early childhood, making it challenging to ensure that the individuals in the comparison group are also experiencing delays or difficulties. This would tend to bias our estimates toward the null. This study does not have any information on privately funded or parent-led interventions or cognitive enrichment activities, which could explain the lack of benefit of EI among individuals whose mothers had high educational attainment and limited benefit among individuals with private insurance. We also have no measures of school characteristics that could contribute to better test scores. We cannot discount the potential for selection bias related to differences among individuals who either leave NYC or attend private schools. This is especially relevant for special education–stratified findings. As shown in the results, children who received EI services but did not receive IEPs by 5 years of age were less likely to enroll in public school for third grade than children who did not receive EI services or an IEP. It is difficult to conclude the direction of any potential bias as there are plausible hypotheses for these children having lower or higher test scores. Last, EI programs have evolved in the last 2 decades and populations within urban communities who access these services may also have changed. In NYC, universal prekindergarten programs and programs for 3-year-olds in certain neighborhoods would also provide other forms of enrichment during early childhood. Updated data linkages can enable routine monitoring of the benefits of EI programming as contexts change.

## Conclusions

The findings of this cohort study suggest that receipt of EI services mandated under Part C of the IDEA act had tangible academic benefits for children. Future research should investigate aspects of EI services among individuals with different diagnoses and delays to increase knowledge on the most beneficial service plans for children with differing needs.

## Notes

Supplement 1. **eFigure 1.** Construction of Analytic Study Population Using Longitudinal Study of Early Development (LSED) 1994-1998 Birth Cohorts **eFigure 2.** Propensity Score Overlap for receipt of Early Intervention Services Before and After Matching Among Children Within the New York City Longitudinal Study of Early Development 1994-1998 Birth Cohort

Supplement 2.
Data Sharing Statement
