# Supplementary material for: Early Intervention Developmental Programming and Childhood Academic Outcomes
Source: JAMA Netw Open. 2026 Feb 9;9(2):e2555890. doi: 10.1001/jamanetworkopen.2025.55890 (PMC12887746; doi:10.1001/jamanetworkopen.2025.55890)
Supplement: Supplement 1. — eFigure 1. Construction of Analytic Study Population Using Longitudinal Study of Early Development (LSED) 1994-1998 Birth Cohorts eFigure 2. Propensity Score Overlap for receipt of Early Intervention Services Before and After Matching Among Children Within the New York City Longitudinal Study of Early Development 1994-1998 Birth Cohort [file jamanetwopen-e2555890-s001.pdf]

# Supplementary Online Content

Stingone JA, McVeigh KH, Lednyak L. Early intervention developmental programming and childhood academic outcomes. *JAMA Netw Open.* 2026;9(2):e2555890. doi:10.1001/jamanetworkopen.2025.55890

**eFigure 1.** Construction of Analytic Study Population Using Longitudinal Study of Early Development (LSED) 1994-1998 Birth Cohorts

**eFigure 2.** Propensity Score Overlap for receipt of Early Intervention Services Before and After Matching Among Children Within the New York City Longitudinal Study of Early Development 1994-1998 Birth Cohort

This supplementary material has been provided by the authors to give readers additional information about their work.

© 2026 Stingone JA et al. *JAMA Network Open*.

**eFigure 1.** Construction of Analytic Study Population Using Longitudinal Study of Early Development (LSED) 1994-1998 Birth Cohorts

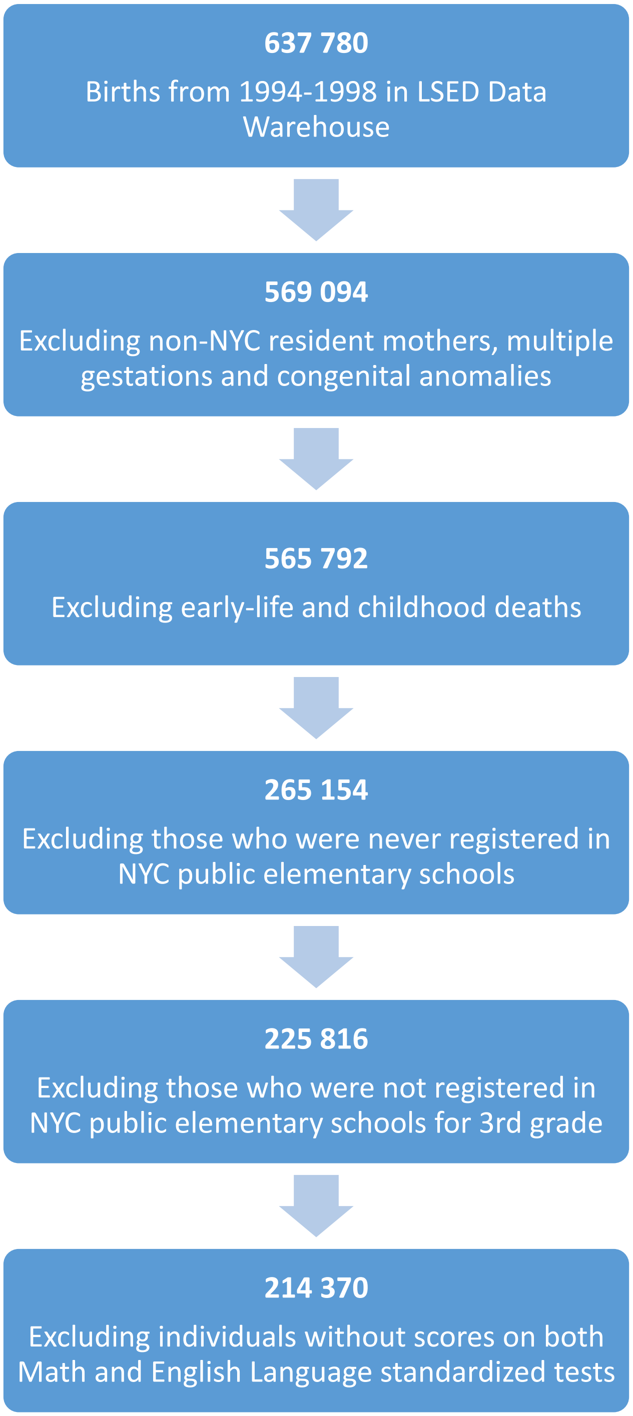


© 2026 Stingone JA et al. *JAMA Network Open*.

**eFigure 2.** Propensity Score Overlap for receipt of Early Intervention Services Before and After Matching Among Children Within the New York City Longitudinal Study of Early Development 1994-1998 Birth Cohort

Plot shows proportion of study population that has specific propensity score. Top panel is before matching, while bottom panel is population after matching.

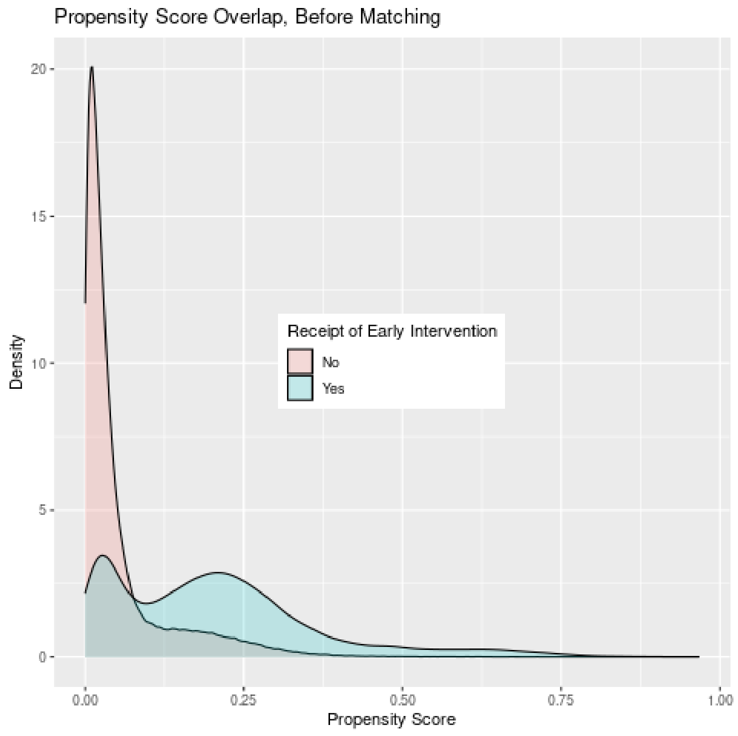


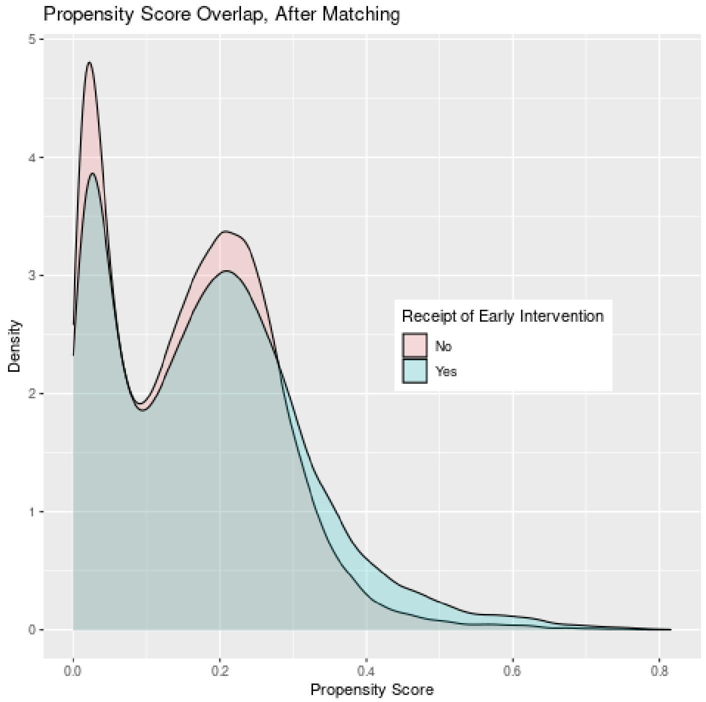


© 2026 Stingone JA et al. *JAMA Network Open*.
