# Supplementary material for: Early Intervention Developmental Programming and Childhood Academic Outcomes
Source: JAMA Netw Open. 2026 Feb 9;9(2):e2555890. doi: 10.1001/jamanetworkopen.2025.55890 (PMC12887746; doi:10.1001/jamanetworkopen.2025.55890)
Supplement: Supplement 2. — Data Sharing Statement [file jamanetwopen-e2555890-s002.pdf]

Data Sharing Statement

Stingone. Early Intervention Developmental Programming and Childhood Academic Outcomes. *JAMA Netw Open*. Published February 09, 2026. doi:10.1001/jamanetworkopen.2025.55890

Data
Data available: No

Additional Information
Explanation for why data not available: Data may be obtained through the New York City Department of Health and Mental Hygiene
